# Detection and Genetic Characterization of Viruses Present in Free-Ranging Snow Leopards Using Next-Generation Sequencing

**DOI:** 10.3389/fvets.2020.00645

**Published:** 2020-09-22

**Authors:** Örjan Johansson, Karin Ullman, Purevjav Lkhagvajav, Marc Wiseman, Jonas Malmsten, Mikael Leijon

**Affiliations:** ^1^Department of Ecology, Grimsö Wildlife Research Station, Swedish University of Agricultural Sciences, Riddarhyttan, Sweden; ^2^Snow Leopard Trust, Seattle, WA, United States; ^3^Department of Microbiology, National Veterinary Institute, Uppsala, Sweden; ^4^Snow Leopard Conservation Foundation, Ulaanbaatar, Mongolia; ^5^Department of Fish and Wildlife Sciences, University of Idaho, Moscow, ID, United States; ^6^Department of Wildlife, Fish, and Environmental Studies, Swedish University of Agricultural Sciences, Umeå, Sweden

**Keywords:** snow leopard, free-ranging, virome, Mongolia, rectal swabs, next-generating sequencing, *Panthera unica*

## Abstract

Snow leopards inhabit the cold, arid environments of the high mountains of South and Central Asia. These living conditions likely affect the abundance and composition of microbes with the capacity to infect these animals. It is important to investigate the microbes that snow leopards are exposed to detect infectious disease threats and define a baseline for future changes that may impact the health of this endangered felid. In this work, next-generation sequencing is used to investigate the fecal (and in a few cases serum) virome of seven snow leopards from the Tost Mountains of Mongolia. The viral species to which the greatest number of sequences reads showed high similarity was rotavirus. Excluding one animal with overall very few sequence reads, four of six animals (67%) displayed evidence of rotavirus infection. A serum sample of a male and a rectal swab of a female snow leopard produced sequence reads identical or closely similar to felid herpesvirus 1, providing the first evidence that this virus infects snow leopards. In addition, the rectal swab from the same female also displayed sequence reads most similar to feline papillomavirus 2, which is the first evidence for this virus infecting snow leopards. The rectal swabs from all animals also showed evidence for the presence of small circular DNA viruses, predominantly Circular Rep-Encoding Single-Stranded (CRESS) DNA viruses and in one case feline anellovirus. Several of the viruses implicated in the present study could affect the health of snow leopards. In animals which are under environmental stress, for example, young dispersing individuals and lactating females, health issues may be exacerbated by latent virus infections.

## Introduction

Infectious diseases can affect the abundance and distribution of animals by reducing survival and reproduction (1, 2). Even small changes in these parameters can substantially increase the extinction risk, for example, in species with slow reproduction or where populations are small (3). This applies to many large carnivore populations as they exhibit a low reproductive output, occur at low densities naturally, and populations are often further reduced and isolated as a consequence of habitat destruction, overexploitation of prey species, and human persecution (4). Reduced survival can also disrupt the social system, where the replacement of dominant males can result in infanticide of young, thus further reducing population numbers (4–6). The detrimental effects that a disease outbreak can cause a felid population are well-exemplified by the canine distemper virus outbreak in Serengeti lions (*Panthera leo*) in 1994. The outbreak, probably originating from domestic dogs (*Canis familiaris*), claimed about 30% of the lion population and is one of the most cited examples of the potential impact that disease can have on felid populations (7). In contrast to the Serengeti lions, which are frequently observed, such an outbreak could occur unnoticed in snow leopards and other less studied felids.

The snow leopard (*Panthera uncia*) is a large felid inhabiting the high mountains of South and Central Asia. The species distribution range covers 1.2–1.6 million km^2^, spanning over 12 countries (8). Snow leopards appear to utilize relatively large territories (9, 10) and occur at low densities (0.9–1.8 adults/100 km^2^) (11). The cold, arid environment inhabited by snow leopards likely has lower microbial abundance than in more temperate and mesic habitats (12). Consequently, snow leopards should encounter disease agents less frequently than many other carnivores and may therefore exhibit lower intrinsic levels of immunity, rendering it vulnerable to disease outbreaks (13, 14). Snow leopards are likely susceptible to most infectious diseases that are known to affect the domestic cat (*Felis catus*), in addition, spill over from other felids and prey animals contribute to the spectrum of infectious diseases that could affect the health of snow leopards (13, 14). Direct routes of transmission are likely most common, these include both intraspecies contact (mating, fighting, socializing) and interspecies contact with wild and domestic prey, other carnivores, and scavengers. To a degree, indirect routes of transmission such as water holes, carcasses, marking sites, and human settlements could also play a role in disease transmission.

Except for a few incidental reports and a recent study focusing on tick-borne bacteria from ticks collected from snow leopards and protozoan infections evidenced by serology (15), there are no published data on infectious diseases in free-ranging snow leopards, owing to their remote and inaccessible habitat, combined with the species' secretive nature. Accordingly, there is very limited information regarding the prevalence and thus potential threat of infectious diseases to snow leopards or which microorganisms are most commonly found in the species (14). Therefore, to begin mapping the occurrence of infectious agents in free-ranging snow leopards, we collected samples from eight animals captured in a telemetry study (16). In the present study, we report viral sequences that were obtained by next-generation sequencing (NGS) of rectal swab samples and, for a few animals, also serum samples from snow leopards in the Tost Mountains of Mongolia.

## Materials and Methods

### Study Area

This study was conducted in the Tost Mountains (43°N, 100°E), a relatively isolated range of mountain massifs (1,600–2,500 m.a.s.l.) in the Gobi Desert in southern Mongolia. Temperatures range from 38°C in the summer to −35°C in the winter, and the annual precipitation is <130 mm of which most falls as rain from June to August. An estimated 10–14 adult snow leopards inhabit the 1,700 km^2^ large area (17) where they prey mainly on ibex (*Capra sibirica*), domestic goats (*Capra aegagrus hircus*), and argali sheep (*Ovis ammon*) (18). The human population consists of ~90 semi-nomadic herder families who move seasonally with their livestock, comprising of ~32,000 goats and sheep (*Ovis aries*), ~1,100 camels (*Camelus bactrianus*), and ~120 horses (*Equus ferus caballus*). In addition, most families have at least one dog that either follow the livestock or roam freely, usually spending the night close to the family's camp. Sympatric predators include gray wolf (*Canis lupus*), Eurasian lynx (*Lynx lynx*), red fox (*Vulpes vulpes*), and marten (Martes spp.).

### Study Animals and Sampling

Snow leopards were captured using modified Aldrich-style foot snares where cats were immobilized with a combination of medetomidine and tiletamine–zolazepam. See reference (16) for a detailed description of capture procedures. We collected rectal swabs from eight snow leopards (three adult males, one subadult male, and four females) from October 2011 to May 2013 by inserting an Amies charcoal cotton swab (Copan Italia S.p.A.) into the rectum and moving it along the wall. The swabs were immediately placed in the charcoal medium. Blood was collected from the cephalic vein and placed in 4 ml blood serum separating tubes (BD vacutainer, Plymouth, UK). Serum tubes stood for 12–15 h to separate cells and serum, which was then decanted into cryovials. We obtained enough serum for NGS from three individuals. In the camp, swabs were stored in a cool and dark box, and sera were kept at −18°C until the samples could be transported to the National Veterinary Institute in Uppsala, Sweden (within 0.5–2.5 months after capture).

### Sample Preparation

Rectal swabs from the eight animals (F3, F7, F8, F9, M1, M7, M9, M10) were immersed in 1,200 μl TE-buffer [100 mM Tris, 10 mM ethylenediaminetetraacetic acid (EDTA), pH 8.0] and homogenized by shaking the tubes vigorously for 1 h at room temperature. After centrifugation (200 g for 5 min), the supernatant was collected and filtered through a 0.45 μm filter to remove particles of bacterium-size and larger. The filtrate was treated with 400 U/ml of DNase I (Roche Applied Science) and 10 μg/ml of RNase A (Invitrogen) in 1× DNase buffer (Roche Applied Science) at 37°C for 2 h to degrade unprotected nucleic acids. Nuclease treatment was also applied to serum samples for three animals (F3, M1, M9) in the same way, and subsequently, DNA and RNA were extracted from 200 μl each of the nuclease-treated samples (the M1 serum sample was also prepared without nuclease treatment) using QIAamp DNA Mini Kit (Qiagen) and a combination of TRIzol (Invitrogen) and RNeasy kit (Qiagen), respectively, following the manufactures' instructions. Sequence-independent single-primer amplification (SISPA) was applied to both RNA and DNA as follows: SuperScript III first-strand synthesis kit (Invitrogen) was used to generate cDNA from the RNA preparation. The reaction was primed by a primer FR20RV-6N (19) following the manufacturer's instructions. Double-stranded DNA was obtained by incubation of the cDNA products with Klenow Fragment DNA polymerase (New England Biolabs) at 37°C for 1 h. Subsequently, the Klenow enzyme was inactivated at 75°C for 10 min. DNA templates were also tagged with the FR20RV-6N primer during a Klenow Fragment reaction at the same conditions. Random amplification of the tagged cDNA and DNA was performed using the primer FR20RV (19) under the following conditions: 10 min at 95°C, followed by 40 cycles of 30 s at 95°C, 30 s at 58°C, and 90 s at 72°C. The reaction was ended with an extra elongation step at 72°C for 10 min. The PCR reaction contained 1× PCR buffer, 2.5 mM MgCl_2_, 2.5 mM dNTPs, 0.4 mM primer, and 1.25 U AmpliTaq Gold DNA polymerase (Applied Biosystems). The amplified DNA fragments were further treated with EcoRV (New England Biolabs) to remove the amplification primers and purified by QIAquick PCR purification kit (Qiagen). Concentration was measured with a Qubit fluorometer using Qubit dsDNA HS (High Sensitivity) Assay Kit (Invitrogen), and a 0.2 ng/μl aliquot was prepared for each sample. Nextera XT DNA Library Preparation Kit (Illumina, Inc.) was used to fragment the input DNA and tag the DNA from each sample with a pair of unique index primers by a 12-cycle PCR amplification. The libraries were purified with AMPure XP beads (Sigma), and Agilent High-Sensitivity DNA Kit (Agilent) was used to verify the length distribution of the fragments and for quantification of the libraries. Finally, an equimolar amount (2 nM) of each sample library with sufficient quality and concentration was pooled, thus constituting two pools of seven serum sample preparations and 15 rectal sample preparations, respectively. The pools were denatured and further diluted to a final concentration of 10 pM. Sequencing was performed on a MiSeq desktop sequencer using MiSeq 500 cycles reagent kit 500 (v. 2) (Illumina, Inc.). Library preparation and sequencing were performed according to the manufacturer's instructions.

### Bioinformatics

The sequence reads were assigned to species by homology searching the NCBI nt database with the BLASTn algorithm as implemented on a Decypher server (TimeLogic®, Carlsbad, CA). Before blasting, the sequence reads were quality checked and trimmed using HTStream v.1.0.0 (HTStream, RRID:SCR_018354) (20). To reduce the computational burden, the trimmed reads were first blasted against the VRL section of the NCBI nt database (i.e., the viral sequences) with a cutoff expect value (*e*-value) of 10^−5^. The reads with BLASTn hits to the VRL database were collected with an in-house python script and subsequently blasted (BLASTn) against the whole nt database with the same e-value. Finally, the reads with best hits to viral sequences in the nt database were collected with an in-house python script. This procedure reduced the computational burden to extract the reads with closest homology to viral sequences in the nt NCBI database with about 90%. These selected subsets of sequence reads were then subjected to *de novo* assembly using the CLC genomics workbench. The reads were mapped back to the assembled contigs, and consensus sequences were extracted by using a low coverage threshold of 5 and a noise threshold of 0.3 (with a secondary nucleotide present above this fraction, a degenerate nucleotide was inserted in the consensus sequence).

The consensus sequences were aligned with related reference sequences for GenBank using a gap open and extension penalty of 10 and 1, respectively. End gaps were free since sequences of different lengths were aligned. Before phylogenetic trees were constructed, the sequences of the alignment were trimmed to the same length. The phylogenetic trees were created using the neighborhood-joining method with the Jukes–Cantor distance measure. The topology was verified using 1,000 bootstrap calculations, and only branches with better than 60% support are shown.

Genotyping of rotavirus A (RVA) were carried out by the reimplementation of the RotaC^2.0^ (21) at the NIAID Virus Pathogen Database and Analysis Resource [Virus Pathogen Resource (ViPR), RRID:SCR_012983] (22, 23).

## Results

Extracted RNA and DNA from eight rectal swabs and three sera were processed for NGS. Only seven rectal samples and three serum samples produced enough sequence reads for further analysis. The samples produced around 2 million sequence reads each. The inference of virus sequences in the snow leopard rectal swabs samples, and sera, was carried out by finding the most closely homologous sequence in GenBank by the blastn algorithm (24). The results are summarized in Table 1. As expected, most of the reads were bacterial despite the experimental procedures to enrich viral sequences in the extracted nucleic acids. The number of viral reads varied in the samples in the range 38–1,844. Generally, mostly DNA viruses were detected with the notable exception of RVA, a double-stranded RNA virus, which was clearly the most abundant virus species.

**Table 1:** Summary of blastn hits to the GenBank nt (database release 228) supported by at least four reads each.

**Animal**	**Sex**	**Collection date**	Rectal swabs and sera (*)		
			**Virus blastn hits**	**No. of reads**	**Min *e*-value**
M1	M	16-10-2011	*Reoviridae* (Rotavirus A)	406	10^−113^
			Virus Chimp162	8	4·10^−61^
			*Circoviridae* (Bat circovirus)	4	2·10^−16^
			*Astroviridae (Mamastrovirus)	191	2·10^−6^
			*Virus Chimp162	58	10^−116^
			*Circoviridae (Bat Circovirus)	7	10^−27^
			*Unclassified *Picornavirales* (Posavirus 1)	6	2·10^−11^
F3	F	18-10-2011	*Reoviridae* (Rotavirus A)	1,049	10^−118^
			Unclassified ssDNA viruses (Sewage-associated circular DNA virus-19)	225	2·10^−18^
			*Herpesviridae* (Human gammaherpesvirus 4; Equine herpesvirus 4)	203	2·10^−43^-8·10^−42^
			Unclassified ssDNA viruses (Circovirus-like genome DCCV-13; Circovirus-like genome RW-E; CRESS DNA virus; *Lake Sarah-associated circular virus-36; *CRESS DNA virus)	13	3·10^−35^-7·10^−10^
M7	M	22-05-2013	Virus Chimp162	106	10^−98^
F7	F	25-04-2012	*Reoviridae* (Rotavirus A)	88	10^−110^
			Virus Chimp 162	14	3·10^−77^
			Unclassified ssDNA viruses (Dragonfly larvae-associated circular virus-3)	9	6·10^−7^
			Unclassified *Picornavirales* (Posavirus 1)	5	10^−8^
			*Herpesviridae* (Equine herpesvirus 4; Suid alphaherpesvirus 1; Epstein–Barr virus; Human herpesvirus 1)	4	10^−8^-5·10^−6^
M9	M	26-10-2011	*Anelloviridae* (Feline anellovirus; Giant panda anellovirus)	20	2·10^−17^-5·10^−9^
			*Anelloviridae* (Torque teno felis virus 2; Torque teno Leptonychotes weddellii virus-1; Paguma larvata torque teno virus; Torque teno didelphis albiventris virus)	15	2·10^−17^-4·10^−6^
			*Herpesviridae* (Human betaherpesvirus 6B)	4	4·10^−29^
F9	F	23-06-2012	*Papillomaviridae* (Feline papillomavirus 2)	108	8·10^−42^
			*Herpesviridae* (Felid herpesvirus 1; Papiine herpesvirus 2; Macaine alfaherpesvirus; Cyprinid herpesvirus 1; Epstein–Barr herpesvirus; Human herpesvirus 7; Ateline alfaherpesvirus; Equid herpesvirus 4)	12	10^−111^-5·10^−6^
			*Poxviridae* (Volepox virus)	6	3·10^−11^
M10	M	18-04-2012	*Reoviridae* (Rotavirus A)	1,635	10^−118^
			*Circoviridae* (Circovirus)	26	10^−121^
			*Genomoviridae/*Unclassified ssDNA viruses (Sewage-associated circular DNA virus-7, 19, 32; Spider-associated circular virus 2; Gemycircularvirus gemy-ch-rat1; Sewage derived gemycircularvirus 2; Lynx canadensis feces-associated genomovirus; CRESS DNA virus)	26	2·10^−87^-2·10^−6^
			Virus Chimp162	8	4·10^−76^
			*Herpesviridae* (Gallid herpesvirus 2; Human herpesvirus 6; Equid herpesvirus 1; Human herpesvirus 7)	6	2·10^−17^-3·10^−6^
			*Astroviridae* (Mamastrovirus)	4	3·10^−6^

*Hits have been grouped according to relatedness. The minimum e-value is the smallest e-value among the reads. For results with several grouped species, the range of minimum e-values is shown. An asterisk indicates results from the serum samples*.

### Rotavirus A

**Figure 1:**
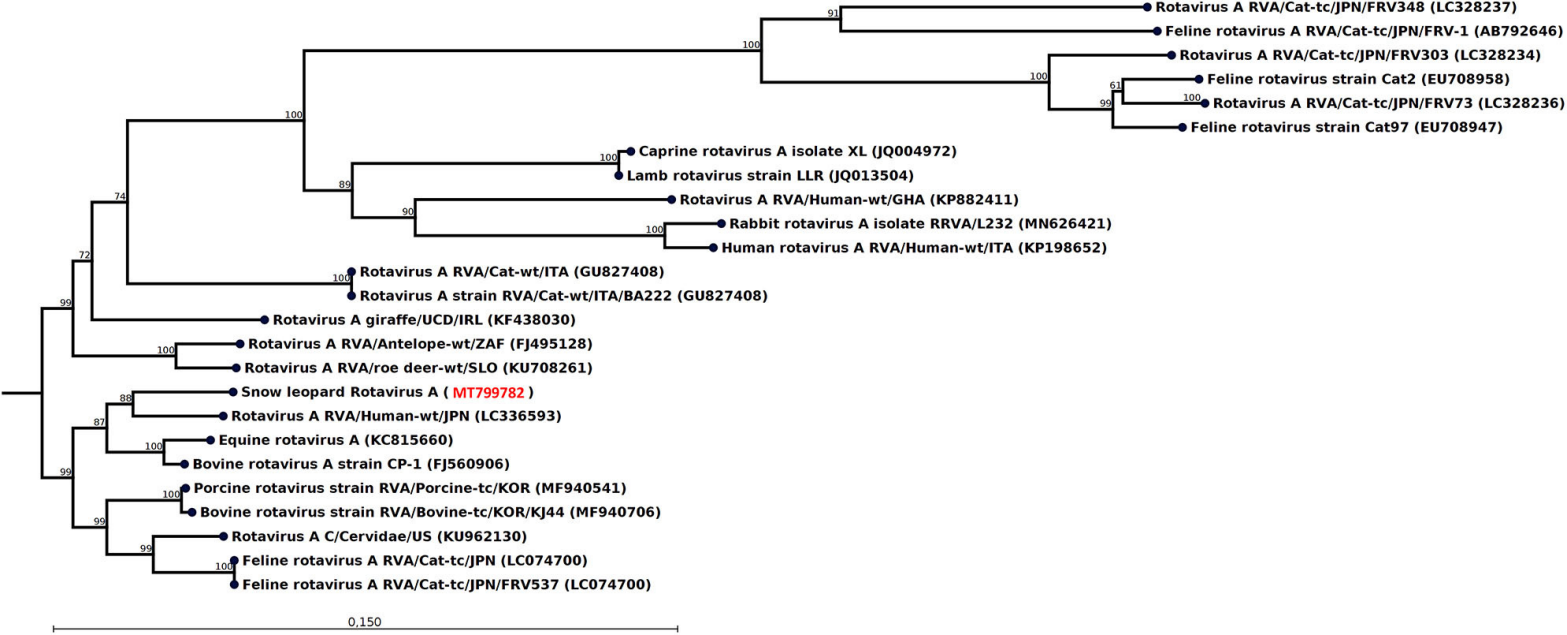
Neighbor-joining phylogenetic tree for a 1,127-nt-long fragment of the rotavirus VP3 gene obtained from the rectal swab of animal M10 together with selected sequences from GenBank (NCBI accessions are given within parentheses). These were selected to include the representative from different hosts with the highest similarity, except that all available isolates from felids were included if not identical. The phylogenetic trees were constructed with the CLC genomics workbench using the neighbor-joining method with the Jukes–Cantor distance measure with 1,000 bootstrap calculations. Only branches with at least 60% bootstrap support are shown. The NCBI accession of the novel snow leopard VP3 sequence is shown in red.

**Figure 2:**
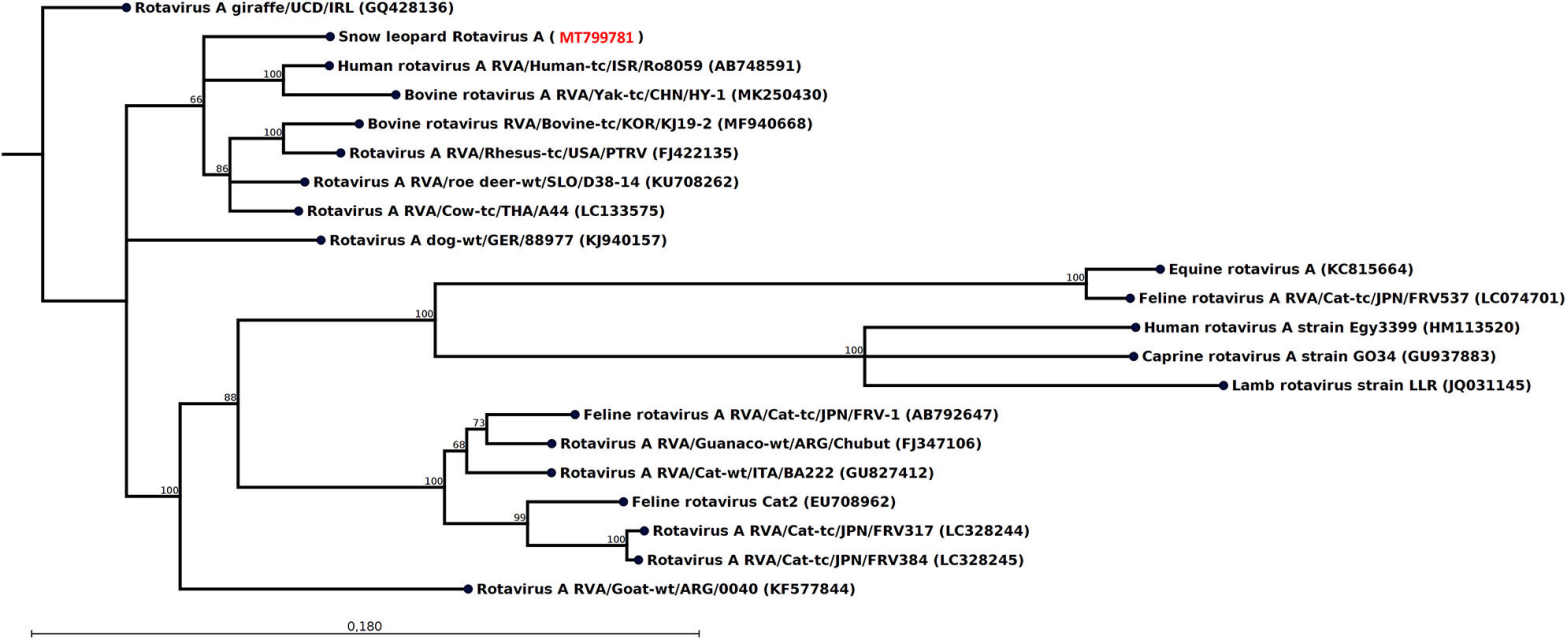
Neighbor-joining phylogenetic tree for a 771-nt-long fragment of the rotavirus NSP1 gene obtained from the rectal swab of animal M1 together with selected sequences from GenBank (NCBI accessions are given within parentheses). These were selected to include the representative from different hosts with the highest similarity, except that all available isolates from felids were included if not identical. The phylogenetic trees were constructed with the CLC genomics workbench using the neighbor-joining method with the Jukes–Cantor distance measure with 1,000 bootstrap calculations. Only branches with at least 60% bootstrap support are shown. The NCBI accession of the novel snow leopard NSP1 sequence is shown in red.

The most striking feature of the rectal swab samples is the large abundance of RVA reads. Four of seven samples (F1, M1, M10, and F7) contain RVA with the number of sequence reads in the range 88–1,635. From animal M1 and M10, a 1,036- and 1,161-nt-long fragment of the NSP1 and VP3 gene, respectively, could be assembled, which allowed genotyping of these fragments. The average read coverage of these contigs was 76 and 223 reads, respectively. After mapping back the reads to the contigs and applying a conservative coverage lower limit of 5 reads, the lengths of the contigs were reduced to 771 and 1,127 nt. These trimmed contigs were used for phylogenetic analysis. The NSP1 contig was of genotype A3 and the VP3 contig was of genotype M2, as determined by the RVA genotyping tool available at the ViPR resource (https://www.viprbrc.org). Of the 11 NSP1 genes from feline RVA available at the ViPR database, the most common genotype was A3 with five members. There were also 11 VP3 genes from feline RVA strains represented at the ViPR resource. Of these, two were of the M2 genotype and the remaining nine were all of the M3 genotype. Phylogenetic analysis of the VP3 fragment shows that the snow leopard fragment is most similar to a human isolate from Japan and is found in a clade with rotaviruses isolated from many different host species, including isolates from primates, bovids, cervids, cats, pigs, and horse (Figure 1). The NSP1 sequence is also found in clade containing rotavirus isolates from humans and bovids (Figure 2). It should be noted that the feline isolates available in GenBank are all isolated from domestic cats, and we have not been able to find rotavirus sequences for comparison from wild felids. In general, the rotavirus sequences found in snow leopard are not related to rotavirus sequences isolated from cats available in GenBank (Figures 1, 2).

### Herpesviruses

Most samples from the seven animals had sequence reads that were similar to various herpesviruses (Table 1). However, few reads with relatively high e-values and blastn hits to herpesviruses found in distantly related host species make most of these hits of questionable relevance. The exceptions are the rectal swab from F9 (Table 1) and the serum sample from M1 (not in Table 1 since only two reads were found), which after assembly of the reads contained a 228- and 289-nt contig, respectively, with sequence identical or closely similar to segments of felid herpesvirus 1 (e.g., GenBank accession MH070348).

### Papillomaviruses

**Figure 3:**
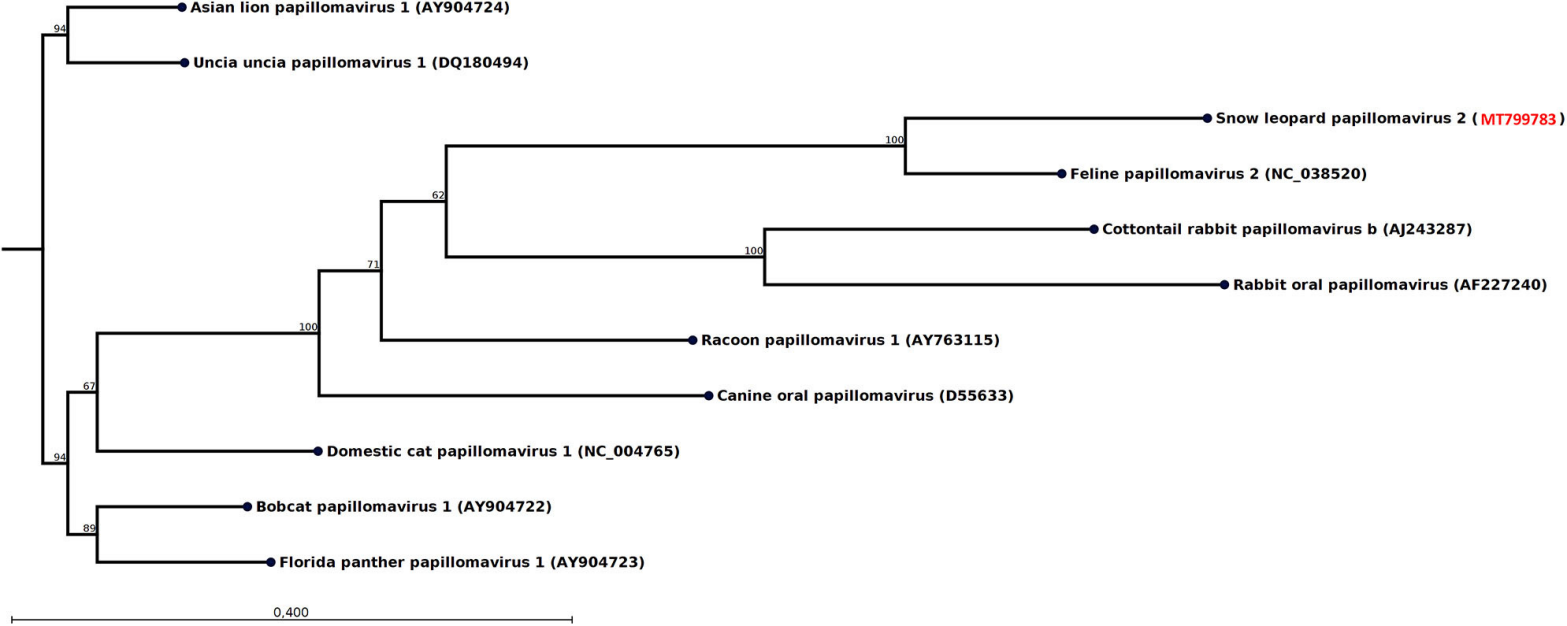
Neighbor-joining phylogenetic tree for a 435-nt-long fragment of the papillomavirus E1 gene obtained from the rectal swab of animal F9 together with selected sequences from GenBank (NCBI accessions are given within parentheses). These were selected to include viral sequences obtained from related host species or for showing high similarity. The phylogenetic trees were constructed with the CLC genomics workbench using the neighbor-joining method with the Jukes–Cantor distance measure with 1,000 bootstrap calculations. Only branches with at least 60% bootstrap support are shown. The NCBI accession of the snow leopard papillomavirus sequence is shown in red.

The swab from one of the female snow leopards (F9) produced 108 reads, which could be assembled into a 489-nt-long contig from the E1 gene most similar to feline papillomavirus type 2 (Table 1). After mapping back the reads on the contigs (average coverage 43 reads) and applying a conservative lower limit for coverage of 5 reads, the length of the contig was reduced to 435 nt. Phylogenetic analysis of this E1 fragment with related papillomaviruses shows that the papillomavirus sequence found in this snow leopard female was most similar to the feline papillomavirus type 2 (Figure 3). The nucleotide identity with feline papillomavirus type 2 (NC_038520) is 74%, and the amino acid identity is 91%.

### Circular Rep-Encoding Single-Stranded DNA Viruses and Anelloviruses

The rectal swabs of four of seven animals (M1, M7, F7, M10) contained viral sequences that were very similar to virus Chimp162 (Table 1). This is a Rep-protein sequence obtained from chimpanzee feces sample collected in Uganda and likely originates from a Circular Rep-Encoding Single-Stranded (CRESS) DNA virus (25). The prevalence of CRESS DNA viruses is further manifested in animal F7 by blast hits to Dragonfly larvae-associated circular virus-3 (26), Lake Sarah-associated circular virus-36 (27), and related viruses in the serum of animal F3 and several hits to the *Genomoviridae* family for animal M10 (Table 1). All these blast hits indicate that CRESS DNA viruses are common in snow leopards and can be found both in the digestive tract and in the blood. Indeed, circoviruses also belong to the CRESS DNA virus group, and the fecal swabs of the snow leopard males M1 and M10 both show evidence of the presence of a circovirus. In the case of M1, sequence reads similar to bat circovirus are found both in the swab sample and in the sera. The fecal swab from the male snow leopard M9 did not show any reads related to CRESS DNA viruses; however, the nucleic acid preparation optimized for DNA viruses showed 35 reads with the highest similarity to the *Anelloviridae* family. The feline anellovirus displayed the closest similarity with an *e*-value of 2 × 10^−17^ (Table 1), which is relatively high, indicating that the putative snow leopard anellovirus only is distantly related to the feline anellovirus.

### Other Viruses

Samples from animal F7 and M1 have sequence reads with homology to porcine stool-associated virus 1 (posavirus 1) (28). The sequence similarity is low as measured by the *e*-values (Table 1), and the number of reads is low. There is thus some evidence that a virus related to posavirus-1 of the *Picornavirales* order may be present in the snow leopards, but this requires further substantiation. This is also true for the reads found with weak homology to astroviruses (animals M1 and M10; Table 1). Astroviruses are frequently found in stool samples from many mammals, and our study suggests that astrovirus may also infect snow leopards, but further investigations are needed to verify astrovirus infections in snow leopards. Six reads are most similar to a volepox virus in animal F9 (Table 1), but the similarity is relatively low, and the finding needs further support. Poxviruses do not normally infect felids, although cowpox is an exception (29). Snow leopards feed on a large array of prey species and can occasionally kill smaller animals such as voles (30), and the volepox virus reads might indicate infected prey animal rather than infection of the snow leopard.

## Discussion

NGS provides a powerful tool for metagenomic investigation and has led to identification of a multitude of novel viruses (31–33). In the present work, we have investigated the intestinal virome of snow leopards by rectal swab sampling and also sera for a subset of the animals. Respiratory viruses are consequently not investigated. In addition, cell-associated viruses of the blood will be detected to a less degree in sera compared to whole blood or buffy coat samples.

The cutoff value for the degree of similarity considered a hit used in the BLAST homology search is set relatively low to make sure that as many potential virus reads as possible are found. This, on the other hand, means that false hits may occur and, if a related virus is absent from the database, the probability for false hits increases. In addition, viruses not infecting the snow leopard but other microorganisms that in turn has infected the snow leopard will also end up as virus hits as well as viruses infecting prey consumed. All these types of presumably false hits have been manually excluded and are not listed in Table 1. The complete list of all virus hits is found in the Supplementary Material.

Reads most similar to RVA are by far the most common viral reads in the fecal swab samples. Many RVA reads were found in animals sampled both in 2011 and 2012, and it appears likely that rotavirus infections are common among snow leopards. Rotaviruses are important enteric pathogens both for humans and animals (34, 35). Reports on rotavirus infections in wild felids are lacking, and the scarce reports in domestic cats have not indicated serious impacts of the disease (36), although exceptions exist (37). In a large epidemiological investigation of RVA in cats housed within 25 rescue catteries across the United Kingdom, only 3% of the 1,727 cats sampled were infected (36). Although only eight snow leopards were sampled, the present study shows that the RVA prevalence of ~67% in snow leopards in the Tost Mountains of Mongolia is surprisingly high compared to existing knowledge. The genotypes of the NSP1 and VP3 genes of RVA could be determined and were found to be A3 and M2. These genotypes have also been found for RVA infecting domestic cats. The significance of RVA infections for the health status of snow leopards remains to be determined. It is known that rotaviruses frequently switch host and can establish productive infections leading to new strains in the novel host (38). It cannot be excluded that the rotaviruses observed here originate from prey either *via* a host switch event or alternatively from feeding on infected prey. The similarity of the snow leopard rotaviral sequences to bovine rotaviruses rather than feline rotaviruses may indicate that.

Herpesvirus is known to occur in wild felids (39) and can in particular affect the health and survival of juveniles (40). Feline herpesvirus type 1 (FeHV-1) is an important cause of acute upper respiratory tract and ocular disease in cats (41). In the present study, a 289-nt-long contig assembled from a serum sample from animal M1 and a 228-nt-long read from the rectal swab from animal F9 were found to be identical or nearly identical to segments from the genome of the FeHV-1 strain KANS_02. This represents to our knowledge the first indication that FeHV-1 circulates among snow leopards.

It was observed some 20 years ago that different patterns were observed with immunohistochemical screening for oral and cutaneous papillomavirus-induced lesions both for snow leopards kept in captivity and domestic cats, indicating the presence of two distinct papillomavirus species (42). The only snow leopard papillomavirus that has been genetically characterized (UuPV-1) is similar to feline papillomavirus type 1 (43). However, the papillomavirus discovered in the present study is more similar to feline papillomavirus type 2 (Figure 3). There is increasing evidence that feline papillomavirus type 2 plays a significant role in the development of skin cancers of domestic cats (44) and could potentially also cause skin cancer among snow leopards.

With application of NGS, a multitude of small circular DNA viruses have been discovered (45–47). For example, many single-stranded DNA (ssDNA) viruses encoding a replication-associated protein have been discovered, and since they by phylogenetic analysis appear to belong to yet uncharacterized but related virus families, they are collectively referred to as CRESS DNA viruses (46, 47). Among the CRESS DNA viruses detected in vertebrates, the *Circoviridae* and *Genomoviridae* are well-established and prevalent. Viruses in the *Parvoviridae* family, which can cause the serious disease feline infectious enteritis (48), have small linear single-stranded DNA genome and thus do not belong to the CRESS DNA virus group. But they have a similar genome organization, and all encode a homologous Rep-protein. Besides the CRESS DNA viruses, also members of the *Anelloviridae* are circular single-stranded DNA viruses commonly found infecting vertebrates. In contrast to the CRESS DNA viruses, anelloviruses lack a Rep-coding gene (49).

In the present study, we observed blast hits with best matches to the putative parvovirus NIH-CQV. However, these viral sequences have been shown to be contaminants from the Qiagen extraction spin columns (50) and will not be further discussed. No other parvovirus blast hits were found.

Similar to many other studies [e.g., (25)] of fecal microbiome, many reads from small circular DNA viruses were observed in the present study (Table 1). In particular, virus Chimp162 (25) is prevalent and is found in four of seven fecal swab samples. Another significant finding of small circular DNA virus is feline anellovirus found in one animal in this study. The clinical significance of these types of viruses has however not yet been well-established.

Finally, it should be noted that few RNA virus reads are found in the present study. The main exception is rotavirus, which is a double-stranded RNA virus known to be very stable and resistant in the environment (51, 52). These observations could be interpreted as the RNA to a large extent has not been well-preserved on the rectal swab samples. The rectal swabs were maintained at ambient temperature during several months at the site of collection before they were transported to locations where they could be kept in freezers. Further sampling taking precautions for RNA preservation, for instance by using PrimeStore^®^ molecular transport medium (Longhorn™ Vaccines and Diagnostics, LLC) or RNAlater™, might be necessary to investigate the presence of other potentially important RNA viruses in snow leopards.

Several of the viruses indicated in the present study such as feline herpesvirus 1, feline papillomavirus 2, and RVA could affect the health of snow leopards (37, 41, 44). The impact that these viruses may have on the health of the snow leopards largely depends on the host immunity, which in turn is modulated by other factors such as availability of prey, suitable habitat, and stress. Thus, in sensitive animals that are under environmental stress, for example, young dispersing individuals and lactating females, any acquired health issues may be exacerbated by latent virus infections, which for example papillomavirus, and herpesvirus are known to establish.

A complication of this study was the isolated location where the animals reside, prohibiting optimal sample maintenance. This fact will bias the viruses observed toward those with DNA genomes and those that possess a more stable capsid structure that protects the nucleic acids better. With these limitations in mind, this is nonetheless a first step toward establishing a baseline characterizing the viral pathogens most commonly found in snow leopards. It is important to continue the sampling in our study area and to expand into other parts of the snow leopard distribution range.

## Data Availability Statement

The datasets presented in this study can be found in online repositories. The names of the repository/repositories and accession number(s) can be found below: https://www.ncbi.nlm.nih.gov, PRJNA626440.

## Ethics Statement

Capture of the snow leopards followed appropriate ethical standards and ethical approval for the study was obtained from Mongolia's Ministry of Environment and Tourism (Government Building II, United Nations Street 5/2, Ulaanbaatar 15160, Mongolia).

## Author Contributions

ÖJ planned the study and carried out the sampling together with PL and JM. KU and MW carried out sample preparations and next-generation sequencing. ML performed bioinformatic analysis of sequence data. ML, ÖJ, and KU wrote the manuscript that was revised by JM and MW. All authors read and approved the final manuscript.

## Conflict of Interest

The authors declare that the research was conducted in the absence of any commercial or financial relationships that could be construed as a potential conflict of interest.
